# Immunity to Pathogens Taught by Specialized Human Dendritic Cell Subsets

**DOI:** 10.3389/fimmu.2015.00527

**Published:** 2015-10-13

**Authors:** Jens Geginat, Giulia Nizzoli, Moira Paroni, Stefano Maglie, Paola Larghi, Steve Pascolo, Sergio Abrignani

**Affiliations:** ^1^Istituto Nazionale di Genetica Molecolare “Romeo ed Enrica Invernizzi” (INGM), Milan, Italy; ^2^Department of Dermatology, University Hospital of Zurich, Zurich, Switzerland; ^3^DISCCO, Department of Clinical Sciences and Community Health, University of Milano, Milan, Italy

**Keywords:** dendritic cells, cytokines, toll-like receptors, T-cell differentiation, cytotoxic T cells

## Abstract

Dendritic cells (DCs) are specialized antigen-presenting cells (APCs) that have a key role in immune responses because they bridge the innate and adaptive arms of the immune system. They mature upon recognition of pathogens and upregulate MHC molecules and costimulatory receptors to activate antigen-specific CD4^+^ and CD8^+^ T cells. It is now well established that DCs are not a homogeneous population but are composed of different subsets with specialized functions in immune responses to specific pathogens. Upon viral infections, plasmacytoid DCs (pDCs) rapidly produce large amounts of IFN-α, which has potent antiviral functions and activates several other immune cells. However, pDCs are not particularly potent APCs and induce the tolerogenic cytokine IL-10 in CD4^+^ T cells. In contrast, myeloid DCs (mDCs) are very potent APCs and possess the unique capacity to prime naive T cells and consequently to initiate a primary adaptive immune response. Different subsets of mDCs with specialized functions have been identified. In mice, CD8α^+^ mDCs capture antigenic material from necrotic cells, secrete high levels of IL-12, and prime Th1 and cytotoxic T-cell responses to control intracellular pathogens. Conversely, CD8α^−^ mDCs preferentially prime CD4^+^ T cells and promote Th2 or Th17 differentiation. BDCA-3^+^ mDC2 are the human homologue of CD8α^+^ mDCs, since they share the expression of several key molecules, the capacity to cross-present antigens to CD8^+^ T-cells and to produce IFN-λ. However, although several features of the DC network are conserved between humans and mice, the expression of several toll-like receptors as well as the production of cytokines that regulate T-cell differentiation are different. Intriguingly, recent data suggest specific roles for human DC subsets in immune responses against individual pathogens. The biology of human DC subsets holds the promise to be exploitable in translational medicine, in particular for the development of vaccines against persistent infections or cancer.

## Introduction

Human beings are constantly exposed to a myriad of pathogens, including bacteria, fungi, and viruses. These foreign invaders or cohabitants contain molecular structures that are sensed by the innate immune system, which mounts a first-line defense and also activates a pathogen-specific, adaptive immune response. The adaptive immune system is composed of B cells that produce specific antibodies, CD8^+^ T cells that can kill pathogen-infected cells, and CD4^+^ T cells that produce effector cytokines and coordinate the immune response. T cells express antigen receptors (T-cell antigen receptors, TCR) that recognize specific peptides presented on MHC molecules. CD8^+^ T cells recognize peptides presented by MHC class-I molecules that are ubiquitously expressed, whereas CD4^+^ T cells are activated by peptide-MHC class-II complexes, which are largely restricted to antigen-presenting cells (APCs). Dendritic cells (DCs) can express very high levels of MHC and costimulatory molecules, and it is generally accepted that they are the relevant cells to induce the activation (“priming”) of antigen-specific “naive” T cells (1, 2) and induce their differentiation into various types of effector T cells.

The elimination or containment of different types of pathogens requires dedicated classes of adaptive immune responses (3). Thus, pathogens like viruses or intracellular bacteria require CD4^+^ and CD8^+^ T cells that produce IFN-γ and kill infected cells (Th1 and CTL, respectively). IL-12 is the critical cytokine that induces this type of response, but IL-12 production by DC is tightly controlled and requires several stimuli derived from pathogens and from CD4^+^ helper T cells (4–9). Conversely, extracellular bacteria and fungi require a different type of response that can be mediated by Th17 cells (10–12). These effector cells are induced by proinflammatory cytokines produced by DC and macrophages (13) and attract neutrophils that in turn phagocytose extracellular bacteria (14). A third type of effector response is the Th2 response, which is required to expel extracellular parasites such as helminths by activating eosinophils and basophils and by inducing antibodies of the IgE class (15). IL-4 is the critical cytokine that induces this response (16), but IL-4 is normally not produced by DC (17, 18). Finally, these different effector responses have to be controlled by specialized regulatory T cells, in particular by IL-10-producing T cells (“Tr1 cells”), which are generated from effector cells and are important to avoid excessive tissue damage by adaptive immune responses (19–22). Cytokines that promote this type of regulatory T-cell response are IFN-α, IL-27, and IL-10 (23–25), and all these cytokines can be produced by DCs (26, 27).

## DCs have the Unique Capacity to Prime T-Cell Responses

Professional APCs have to present pathogen-derived peptides on MHC molecules to activate antigen-specific T cells. DCs are phagocytic in the immature state, i.e., under steady-state conditions and upon initial pathogen encounter, and can take up antigenic material by pinocytosis or by surface receptor-mediated internalization (28). Proteins from pathogens are then shuttled to lysosomes where they are chopped to peptides and loaded on MHC class-II molecules (29, 30). These peptide–MHC complexes are then transported to the plasma membrane to activate specific CD4^+^ T cells. The presentation of peptides derived from exogenous proteins on MHC class-I, a process called cross-presentation (31, 32), is a largely unique feature of DCs and is particularly important to activate CD8^+^ T cells in viral infections. Virus-infected cells express viral proteins in the cytosol where they are degraded to peptides by the proteasome, translocated to the endoplasmic reticulum by TAP proteins, and loaded on MHC class-I molecules (31). However, since DCs are not necessarily infected by viruses, they must be able to process virus-derived proteins also from external sources, such as virus-infected cells, to activate CD8^+^ T cells. The mechanism of cross-presentation is still incompletely understood, but two distinct pathways via vacuoles and peptide translocation from phagolysosomes to the cytosol have been described (32). It is believed that cross-presentation is the most important pathway leading to the induction of cytotoxic T-cell responses, and excellent reviews have been published on this relevant topic (31–33).

Naive T cells have a very high activation threshold (34), and only professional APCs that express high levels of MHC and costimulatory molecules such as DCs are able to induce proliferation of naive T cells (35). Several receptor–ligand interactions contribute to naive T-cell activation (36–38), but CD28 costimulation is particularly important to amplify the signal transduced by the TCR (39). Monocytes efficiently present peptides derived from extracellular proteins on MHC class-II to activate antigen-experienced CD4^+^ T cells (34), and this capacity can be exploited to selectively expand antigen-specific memory T cells (40). However, monocytes have an approximately 1000-fold lower capacity to prime naive CD4^+^ T cells as compared to DCs (Nizzoli et al., under review) and home to non-lymphoid tissues in the steady state. However, upon inflammation, they can differentiate to inflammatory DCs (41) and home to lymph nodes where they can activate T cells (42, 43). In addition, there is some evidence that CD16^+^ subsets of human blood monocytes might contain DCs (27, 44, 45). Naive T cells constantly recirculate in the blood and migrate through secondary lymphoid organs (46), but are largely excluded from non-lymphoid tissues. In secondary lymphoid tissues, they migrate to the T-cell zone, where they encounter DCs (47). B cells are also present in secondary lymphoid organs and can potently present antigen to T cells when they internalize and process antigens that have specifically bound to their B-cell receptor (48). However, B cells are physically separated from naive T cells in lymph nodes and only following TCR activation naive T cells migrate to the B-cell zone where they interact with antigen-specific B cells to induce antibody production (49, 50). Thus, antigen presentation by B cells appears to be important for the activation of antigen-experienced T cells rather than for naive T-cell priming.

## Pathogen-Associated Molecular Patterns Induce DC Maturation

Dendritic cells are generated from committed precursors in the bone marrow that are released into the circulation to seed peripheral organs (51–55). Both monocytes and DCs can be derived from common myeloid progenitors (CMPs), but committed precursors that selectively give rise to monocytes or DCs (51) or even selected DC subsets (53, 54) have been identified in humans and mice. DCs are poorly stimulatory in the immature state and can induce a partial T-cell activation, leading to deletion of autoreactive CD8^+^ T cells (56–59). In addition, they promote self-tolerance by inducing Foxp3^+^ regulatory CD4^+^ T cells that suppress autoreactive T cells (60). Pathogens induce the maturation of DCs that consequently acquire the capacity to produce polarizing cytokines and to prime pathogen-specific effector T-cell responses. Pathogen-derived molecular patterns [PAMPs (61, 62)] are recognized by DCs and lead to the efficient presentation of antigens to T cells (63). There are different classes of pathogen-sensing receptors, including Toll-like receptors (62, 64), nucleotide-binding oligomerization domain (NOD)-like receptors (65), retinoic acid-inducible gene 1 (RIG-I)-like receptors (66), and C-type lectins (67). TLRs recognize different PAMPs, including nucleic acids or cell wall components such as proteins and lipoproteins (68, 69). In the case of viruses, nucleic acids are sensed not only by different TLRs in endosomes but also by cytosolic receptors like RIG-I (66, 70) and induce a potent activation of DCs. Importantly, subsets of DCs express different patterns of pathogen-sensing receptors and might thus preferentially respond to individual pathogens (71, 72). DNA viruses such as cytomegalovirus (CMV) and herpes simplex virus (HSV) and also bacteria can activate DCs via unmethylated CpG-containing DNA (69), which is sensed by TLR9. Double- and single-stranded RNAs, which are generated by both DNA and RNA viruses, are sensed by DCs via TLR3 (73) and TLR7/8 (74, 75), respectively. Of note, TLR3 is restricted to mDCs (71) and induces cross-presentation capacities (76). Viruses such as respiratory syncytial virus (RSV) and hepatitis C virus (HCV) can also activate DCs via TLR2 or TLR4, which are expressed on the plasma membrane and recognize viral proteins (77). TLR2 is also involved in immune responses to fungi (78) and Gram-positive bacteria (79, 80) while TLR4 recognizes lipopolysaccharide (LPS) (81), a cell membrane compound of Gram-negative bacteria. Many pathogens like viruses activate DCs via multiple TLRs (77). Moreover, other immune cells, including T cells themselves, feed-back on DCs to regulate the ongoing response. In particular, CD40 stimulation by CD4^+^ helper T cells is crucial for CD8^+^ T-cell stimulation and IL-12 production (4, 5). Moreover, IFN-γ (6) and paradoxically also IL-4 (7, 8) that can be provided by T cells further enhance IL-12 production (9).

Surface TLRs such as TLR2 and TLR4 signal via the adaptor protein Myd88 (82) to induce the activation of Map kinases and the nuclear translocation of the transcription factor NF-κB, which in turn induces the transcription of proinflammatory cytokines (62). Endosomal TLRs 7, 8, and 9 also signal via Myd88 but activate IRF7, which in turn induces type-1 interferon production (83, 84). TLR3 is an exception since it does not signal via Myd88 but utilizes TRIF (85) to activate IRF3 (86, 87) or IRF7 (88). How all these complex signaling pathways are integrated by DCs to induce the appropriate T-cell response is still incompletely understood (88–90).

## Specialized DC Subsets Induce Different Classes of T-Cell Responses in Mice

Dendritic cells in mice can be subdivided into distinct subsets with specific functions. Some DCs are stably resident in lymph nodes while others are positioned in non-lymphoid tissues to sense tissue-invading pathogens, but are migratory and are recruited via the lymph following pathogen encounter in a CCR7-dependent manner (91, 92). In secondary lymphoid tissues, two major DC subsets are myeloid DCs (mDCs) and plasmacytoid DCs (pDCs; Table 1) (72, 93, 94). Both pDCs and mDCs upregulate MHC and costimulatory molecules like CD80 and CD86 upon maturation (72) that bind to CD28 and are required to induce full T-cell stimulation (39). However, pDCs are poorly phagocytic and have a different regulation of MHC class-II turnover upon maturation as compared to mDCs (95). Thus, mDCs stop phagocytosis and peptide loading on MHC upon pathogen recognition and stably present peptides from antigenic material they had acquired upon pathogen encounter (30, 96, 97). This maturation-induced stabilization of peptide–MHC complexes enhances the priming of pathogen-specific T cells by mDCs. In contrast, pDCs continue to present new peptides on MHC complexes even in the mature stage (95). On the one hand, this limits their capacity to stimulate pathogen-specific T cells; on the other hand, this enables them to present also late-expressed viral antigens when they are actively infected. This diverse regulation of MHC–peptide stability in mDCs and pDCs suggests that they present different antigens to T cells.

**Table 1:** **Surface markers expressed on human and mouse DC subsets**.

Subsets	Mouse (spleen)			Human (blood)		

	CD8α−	CD8α+	pDC	mDC1	mDC2	pDC
CD11c	+	+	Low	++	+	−
CD11b	+	+/−	−	+	−	−
BDCA-1/CD1c	n/a	n/a	n/a	+	−	−
BDCA-2/CD303	−	−	+	−	−	+
BDCA-3/CD141	−	−	−	−	+	−
BDCA-4/CD304	−	−	−	−	−	+

*This table summarizes the expression of widely used surface markers to identify DC subsets in humans and mice*.

**Figure 1:**
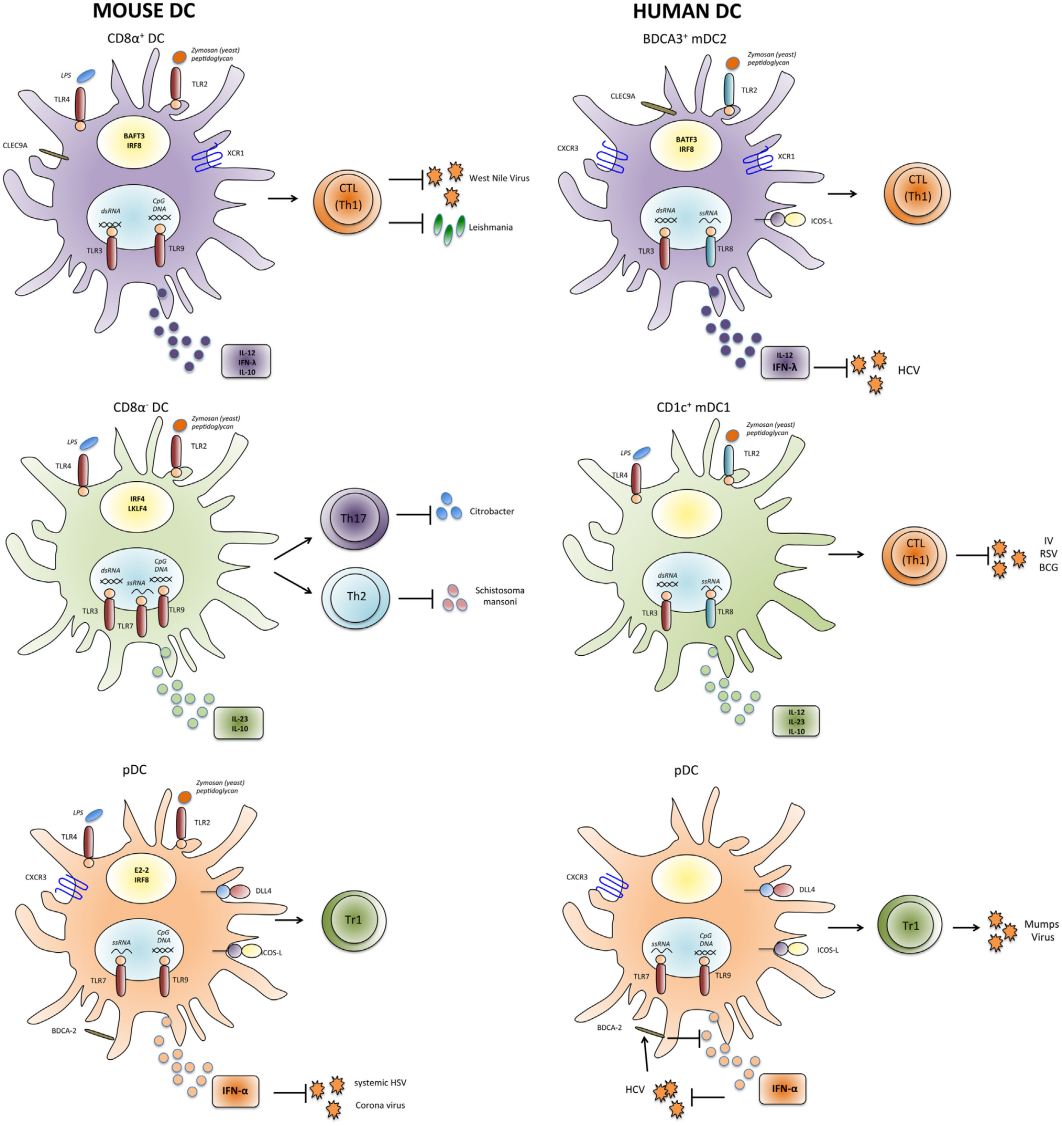
**Properties and functions of human and mouse DC subsets**. Human and mouse mDC and pDC subsets express partially different patterns of pathogen-sensing receptors and cytokines and might thus have unique functions in inducing appropriate types of T-cell responses against individual pathogens. IV, influenza virus; HCV, hepatitis C virus; RSV, respiratory syncytial virus; HIV, human immunodeficiency virus; HSV, herpes simplex virus; BCG, Bacillus Calmette–Guérin.

Plasmacytoid DCs are present in lymph nodes and are largely absent from non-lymphoid organs, but they can be recruited upon inflammation (98). The role of pDC in T-cell priming is still debated (99). There is consensus that they are poorly stimulatory in their resting state (100, 101), but while some groups proposed that they become potent APCs following TLR stimulation and prime CD4^+^ and cross-prime CD8^+^ T-cell responses (102–105), others concluded that also mature pDCs have only low priming and cross-priming capacities and might rather be tolerogenic (101). The rapid and abundant production of type-1 interferon by pDC suggests a pivotal role in viral infections, even if their capacity to prime virus-specific T cells directly appears to be limited. IFN-α can also be produced by other immune cells and by virus-infected cells, but the early and systemic IFN-α response is believed to depend on pDCs (101). Consistently, in the case of HSV infections, it was shown that pDCs were important for systemic but not local protection (106). However, in several other viral infections in mice, including vesicular stomatitis virus (VSV), lymphocytic choriomeningitis virus (LCMV), RSV, and mouse cytomegalovirus (MCMV), pDCs do not seem to play a major role (100). In marked contrast, in mouse hepatitis virus (MHV) infection, the antiviral response against this coronavirus was largely pDC dependent (107) (Figure 1). Finally, pDCs have been found by several groups to induce the production of the anti-inflammatory cytokine IL-10 by CD4^+^ T cells, suggesting that they might be important to inhibit excessive T-cell responses. Several proteins expressed by pDCs were found to promote IL-10 induction in T cells, including the Notch ligand Delta-like 4 (108), ICOSL (109, 110), as well as IFN-α (23, Nizzoli et al., under review).

Myeloid DCs are a heterogeneous population, and different mDC subsets can be identified that preferentially initiate different types of adaptive immune responses (Figure 1). In the spleen of mice, mDCs can be subdivided into CD8α^+^ and CD8α^−^ subsets (Table 1). CD8α^+^ mDCs produce high levels of bioactive IL-12p70 and efficiently cross-prime CD8^+^ T-cell responses (111). They express CLEC9A, a C-type lectin, that enables them to take up antigenic material from dying cells, and their generation was shown to rely on the transcription factors BATF3 and IRF8 (112, 113). Moreover, they express the chemokine receptor XCR1 that favors their colocalization with CD8^+^ T cells (114). Altogether the present evidence indicates that CD8α^+^ DCs are specialized to induce Th1 and CTL responses in response to intracellular pathogens (115, 116). Notably, DCs in the gut that express CD103 have similar characteristics and are closely related to CD8α^+^ DC (117, 118). CD8α^−^ DCs express CD11b and can be further subdivided into CD4^+^ and CD4^−^CD8^−^ subsets. They preferentially prime CD4^+^ T-cell responses (119) and promote Th17 responses, but they can also induce Th2 cells (113, 120). Interestingly, CD11b^+^ DCs produce IL-23 in the gut and are required for protection against *Citrobacter rodentium* (121). Their generation depends on the transcription factor IRF4, while KLF4 expression is required for Th2, but not for Th17 induction (122). Notably, however, CD8α^−^ DCs and also pDCs can cross-prime CD8^+^ T-cell responses under certain conditions (102–104, 123). Moreover, it was shown that upon appropriate microbial stimulation all mDC subsets have the potential to promote either Th1 or Th2 responses (124). Thus, although the proposed functional specialization of DC subsets is an intriguing and helpful concept, it might also be an oversimplification, since DC subsets have considerable plasticity and the induction of a specific type of immune response critically depends on the stimuli they receive from pathogens as well as from other immune cells (125).

## Different Pathogen Sensing by DC Subsets in Humans and Mice

High numbers of human DCs can be generated *in vitro* by culturing monocytes with cytokines (41), and the large majority of studies on human DCs have been done with these monocyte-derived DCs. They are primary cells and show many behaviors of *in vivo* occurring DCs, including cytokine production as well as stable and potent antigen presentation upon maturation with TLR ligands (125). However, monocyte-derived DCs are not the appropriate model to study the role of specialized DC subsets in human immune responses.

Dendritic cells circulating at low frequency in human peripheral blood share several features with murine splenic DC subsets (126) (Table 1). Human pDCs have been identified more than 15 years ago as the natural IFN-α-producing cells (127, 128). They express TLR7 and TLR9 and produce large amounts of IFN-α in response to CpG DNA or influenza virus. Similar to their murine counterparts, they are poorly stimulatory (94), express the C-type lectin BDCA-2 (93), and induce IL-10 production in CD4^+^ T cells (129). In addition, subsets of mDCs can also be found in human blood and in tissues (130–133). As their murine homologues, they express CD11c and potently prime CD4^+^ and CD8^+^ T-cell responses. The expression of CD1c/BDCA-1 and CD141/BDCA-3 identifies two subsets among human mDCs in peripheral blood (93) and also in secondary lymphoid organs (105, 132, 134, 135). BDCA-3^+^ “mDC2” (Table 1) are rare, but it could recently be demonstrated that they represent the human counterpart of murine CD8α^+^ DCs (136–140). Thus, as CD8α^+^ DCs, they selectively express CLEC9A and XCR1 and are dependent on the transcription factor BATF3 (112, 136, 138, 140, 141). Importantly, they can cross-present exogenous antigens on MHC class-I to CD8^+^ T cells and produce IL-12 (134–136). CD1c^+^ “mDC1” (Table 1) are more frequent and share some features with CD8α^−^ DC, including CD11b expression and IL-23 production (121, 142, Nizzoli et al., under review). Also TLR3 expression in DC subsets appears to be similar in humans and mice, since it is expressed at high levels on CD8α^+^ DCs and mDC2, at lower levels on CD8α^−^ DCs and mDC1, and absent on pDC. Surprisingly, TLR3 in mice is not required for immune responses against several viruses, including LCMV, VSV, MCMV, and Reovirus, suggesting that TLR3 has not a pivotal role in antiviral immune defense (143). Consistently, TLR3 deficiency in humans selectively leads to uncontrolled HSV1 infections in the central nervous system (CNS) (144).

Different subsets of DC have also been identified in human non-lymphoid tissues where they are strategically positioned to recognize invading pathogens, in particular at barrier surfaces. These migratory DC subsets play a crucial role to transport antigenic material of pathogens that invade specific tissues to draining lymph nodes and thus to initiate a tissue-specific T-cell response (130, 145, 146). Human Langerhans cells were first described more than a century ago and reside in the epidermis and are thus the first DCs that encounter skin-invading pathogens. Upon activation, they mature and migrate to draining lymph nodes to activate CD4^+^ and CD8^+^ T cells. In the dermis, different subsets of interstitial DCs are present and can be classified according to CD14, CD1a, and CD141 expression. Dermal CD14^+^ cells might represent monocyte-derived macrophages rather then DCs (147), but CD1a^+^ and CD141^+^ DCs, respectively, resemble the CD1c^+^ and CD141^+^ DC subsets in peripheral blood (148). Also in the lung and the liver, DC subsets that are related to CD1c^+^ and CD141^+^ DCs could be identified (133). Finally, in the human intestine, DC subsets that express CD11b and CD103 are similar to CD1c^+^ and CD141^+^ DCs, respectively, and these intestinal DC subsets are also largely conserved between humans and mice (149).

Although the similarities between human and mouse DC subsets are often emphasized, there are also some important differences in pathogen sensing by DCs in humans and mice (150). Importantly, the expression of several relevant TLRs is not conserved (Figure 1), presumably because humans and mice have evolved under the selective pressure of different pathogens. Thus, in mice, TLR7 and TLR9 are expressed by both pDC and mDC subsets (71), whereas in humans, they are restricted to pDCs (72). Also TLR4 expression is more restricted in human DCs, since it is expressed by mDC1 but not by mDC2 (136). Moreover, TLR8 is not expressed by human pDCs (72), and some agonists of human TLR8 such as the resiquimod R848 do not activate murine TLR8 (75, 151). Another relevant difference seems to be the role of the adaptor protein Myd88, which transduces signals from all TLRs with the notable exception of TLR3. Thus, mice deficient for Myd88 are highly susceptible to several infections by bacteria, viruses, parasites, and fungi. Conversely, Myd88-deficient patients are selectively affected by infections with pyogenic bacteria in childhood (152). Finally, human CD1c^+^ DCs and also Langerhans cells seem to have superior capacities to cross-present antigens and to induce CTL responses as compared to their murine homologues (105, 134, 153, 154). Overall, these differences in pathogen sensing and T-cell activation between human and murine DCs are likely to have an important impact on their role in immune responses against specific pathogens.

## Subset-Specific Cytokine Production by Human DCs

Dendritic cell subsets in humans and mice express not only different patterns of toll-like receptors, but they have also partially distinct cytokine profiles (Figure 1). In particular, human mDC1 have a complex and quite unique regulation of cytokine production. Thus, while LPS triggers only low levels of cytokine production by mDC1, dual TLR stimulation with LPS or Poly-I:C (TLR3 ligand) in combination with R848 induces very high levels of a broad panel of cytokines, including TNF, IL-6, IL-10, IL-12, and IL-23 (Nizzoli et al., under review). The very potent cytokine-producing capacity of mDC1 has been missed in several studies where mDC1 were activated with single TLR ligands (45, 155, 156). Of note, single TLR stimulation is sufficient to induce antiviral cytokines by mDC2 and pDCs (see below) and proinflammatory cytokines by monocytes. Although mDC1 can secrete several proinflammatory cytokines that promote Th17 cell generation including IL-23 (142), it is unclear if they are the physiological inducers of Th17 cells or if monocyte-derived, inflammatory DCs do the job (12, 157). Also the identity of the DC subset that induces human Th2 responses is still enigmatic. It was originally proposed that mDCs induce Th1 polarization and pDCs Th2, but later it was shown that also pDCs can drive Th1 responses (158, 159). More recently, mDC2 but not mDC1 were found to induce Th2 cells in an aberrant response to influenza virus (160).

In apparent contrast to CD8α^−^ DCs, mDC1 can produce high levels of IL-12 (134, 135), suggesting a relevant role in immune responses against intracellular pathogens. Moreover, the production of the anti-inflammatory cytokine IL-10, which can be produced by all mDCs in mice, is largely restricted to mDC1 in humans (Nizzoli et al., under review). Stimulation of mDC1 with the intestinal bacterium *Escherichia coli* or with LPS alone induces IL-10 and was proposed to induce a tolerogenic state in mDC1 (155). Although IL-10 is indeed a tolerogenic cytokine and a well-established negative regulator of DC maturation and cytokine production (161), it can paradoxically also have positive effects, in particular on CD8^+^ T-cell responses (162, 163). Consistently, we found that IL-10 produced by mDC1 completely blocked the cross-priming of low-affinity CTL and enhanced the responsiveness of CD8^+^ memory T cells to the homeostatic cytokine IL-15. Thus, mDC1-derived IL-10 appears to play an important positive role in CTL responses, since it selects high affinity cells upon priming and inhibits CTL memory attrition at the same time (Nizzoli et al., under review).

While mDC1 can secrete a broad panel of pro- and anti-inflammatory cytokines, mDC2 and pDC are largely dedicated to secrete high levels of antiviral cytokines. The subset-specific production of IFN-α by pDC (128) and of IFN-λ by CD8α^+^ and mDC2 (134, 137) appears to be largely conserved between humans and mice. The very potent IFN-λ-producing capacities of BDCA-3^+^ DC (134, 137) suggest that analogous to pDCs they might be the relevant source of early and systemic IFN-λ in viral infections. Notably, IFN-λ has antiproliferative and antiviral activities similar to type-I interferon, but the expression of the IFN-λ receptor is much more restricted and found mainly on epithelial cells at barrier surfaces and in the liver (164). MDC2 can also secrete selected isoforms of IFN-α (165) and some IL-12 (134–136, 138), consistent with the view that they play an important role in antiviral immune responses. As previously mentioned for murine pDCs, IFN-α is not only a powerful antiviral cytokine that activates several different types of immune cells, but it also induces IL-10 production in CD4^+^ T cells, suggesting that pDCs induce Tr1-like regulatory T cells also in humans (21, 23, 108, Nizzoli et al., under review).

## Specific Roles of Human DC Subsets in Responses to Individual Pathogens

The more restricted expression of TLRs and the specific cytokine-producing capacities of human DC subset suggest that they play unique roles in immune responses against individual pathogens. The roles of human DC subsets in pathogen-specific immune response are however difficult to address directly because patients that selectively lack a DC subset of interest have not been identified so far. Nevertheless, some interesting findings were reported. In particular, mDC2 appear to be highly relevant in HCV infection. Single-nucleotide polymorphisms in the IFN-λ3 gene locus are strongly associated with spontaneous clearance and response to therapy in HCV patients (166). All DC subsets can secrete some IFN-λ1 (134, 167), but mDC2 produce much higher amounts. Moreover, IFN-λ2/3 are largely restricted to mDC2, and importantly HCV induces IFN-λ3 production by mDC2 (168, Nizzoli et al., under review). Thus, mDC2 appear to be a highly relevant source for protective IFN-λ3 in HCV infection (169). Interestingly, an important role for mDC1 rather than for mDC2 was recently proposed in tuberculosis (170, 171). Thus, mDC1 were more efficiently infected with the Bacillus Calmette–Guérin (BCG) vaccine than other DCs and induced the activation of pDCs and CD8^+^ T cells. Notably, mDC1 could not be replaced by mDC2 in this system, suggesting that mDC1 could play a non-redundant role in the defense against selected intracellular pathogens. MDC1 and mDC2 have also been suggested to play different roles in RSV infection (172, 173). Thus, mDC subsets produced different cytokines in response to RSV, consistent with their different cytokine profiles upon stimulation with purified TLR ligands (134, Nizzoli et al., under review). Moreover, they induced different classes of T-cell responses, with mDC1 inducing preferentially Th1 cells and mDC2 inducing predominantly Th2 and T-regulatory cells. Similarly, mDC2, but not mDC1, were found to induce Th2 response to influenza virus (160). Also the capacity of pDCs to induce IL-10-producing regulatory T cells has been documented with a relevant pathogen, since pDCs were shown to induce IFN-γ and IL-10 production in antigen-experienced CD4^+^ T cells specific for mumps virus (129). Conversely, CD11c^+^ mDCs, which contain both mDC1 and mDC2, induced IFN-γ and, surprisingly, IL-5.

It is largely accepted that pDC-derived IFN-α is important to contain human viral infections. Thus, stabilized pegylated IFN-α is a widely used therapy for HCV patients. IFN-λ appears to be similar effective, but is less toxic presumably because of the more restricted expression of its receptor (164). Interestingly, the HCV glycoprotein E2 is a ligand for BDCA-2, which is specifically expressed on pDCs (Table 1) and inhibits IFN-α production (174, 175). In this way, HCV might inhibit IFN-α production to establish chronic infection. Finally, pDCs are also targeted by human immunodeficiency virus (HIV), but whether they play a protective or detrimental role is still unclear (176).

## Exploiting DC Biology: Vaccines that Induce Humoral and Cellular Immune Responses

Vaccines have been a major breakthrough for human health. Attenuated or killed pathogens are highly efficient to induce protective cellular and humoral immune responses, and the induced protective memory can last for a lifetime (177, 178). However, since these pathogen-based vaccines also have considerable side effects, proteins in combination with adjuvants that activate APCs are more often used. Protein vaccines induce CD4^+^ T-cell responses and neutralizing antibodies, but they are poorly efficient in inducing cytotoxic T-cell responses and are also rather inefficient in inducing Th1 cells (179, 180). Frequently used adjuvants are alum, oil-in-water emulsions like MF59, and more recently also monophosphoryl lipid A (MPL), a detoxified form of LPS. In mice, different adjuvants were shown to induce different proinflammatory cytokines. Thus, alum acts via uric acid on inflammatory DCs (181), which leads to NOD-like receptor protein-3 (NALP3)-dependent IL-1β production (182). Conversely, MPL does not induce IL-1β (183) but induces specific antibodies through an IL-6-dependent mechanism (184), while MF-59 and alum act independently of IL-6 (185). However, the different TLR expression and cytokine production by human APC subsets should be considered when translating this knowledge from animal models to patients. A recent interesting report analyzed the response of APC subsets to 13 different vaccines and concluded that different vaccines activate indeed different APC populations (186). More direct information on the effect of DCs was obtained by vaccinations with peptide-pulsed monocyte-derived DCs in cancer patients, which can induce tumor-specific CD8^+^ T cells (187), but the clinical responses were so far largely insufficient. MDCs might be more potent and are currently tested in clinical trials.

Nucleic acid-sensing TLRs are particularly potent to induce CD8^+^ T-cell responses in mice (188) and have recently been employed as adjuvants in vaccines. Examples are CpG-DNA that stimulates TLR9 (189), and the TLR7 ligand imiquimod, which is used as a cream to stimulate DC locally in the skin, and was shown to induce CD8^+^ T-cell responses *in situ* (190). Vaccines consisting of plasmid DNA coding for relevant protein antigens are a novel approach that efficiently induces humoral and cellular immune responses in animals. However, in humans, these DNA vaccines are often poorly immunogenic (191), presumably because they have only low adjuvant activity and stimulate mainly cytosolic DNA sensors rather than TLR9 (192), which in addition is restricted to pDCs and B cells in humans. An alternative promising approach is the vaccination with mRNA (193, 194), which delivers not only the antigenic protein directly to the cytosol, thereby bypassing the requirements for cross-presentation (195), but also induces mDC and pDC maturation and cytokine production via TLR7/8 at the same time (196). Indeed, intradermal injection of naked mRNA results in local uptake and translation of the nucleic acid (197) followed by the development of an adaptive immunity in mice (198) and in humans (199, 200). Since also lymph node-resident DCs are expected to be appropriate APCs to process antigens encoded by mRNA, direct injection of nucleic acid into lymph nodes has also been evaluated. In animal models, intra-lymph node injections of mRNA result in expression of the protein encoded by the mRNA in DCs. Furthermore, the injected mRNA activated lymph node-resident APCs and induced potent CD4^+^ and CD8^+^ T-cell responses as well as prophylactic and therapeutic antitumor immunity (201). The approach is currently being evaluated through two clinical studies exploring the efficacy of intra-lymph node mRNA vaccination in advanced melanoma patients. As a further development, systemic administration of a liposomal formulation of mRNA that delivers the nucleic acids to APCs present in secondary lymphoid organs is also being evaluated. Using the functional diversity of DCs *in vivo*, and their specific capabilities in generating appropriate adaptive immune responses, those systemic synthetic vaccines might recapitulate the natural mechanisms of immunity developed during pathogen infection and guarantee the development of therapeutically efficacious immune responses.

## Conclusion and Perspective

Dendritic cells continue to attract much interest of immunologists because they are the most potent APCs in the immune system and are the principal inducers of naive T-cell differentiation. Intensive research in the last years has established that different subsets of DC exist in mice that have specialized functions and preferentially induce different types of immune responses. In humans, much has been learned from *in vitro* differentiated monocyte-derived DCs, and more recently, also different subsets of DC populating human tissues have been analyzed at the molecular and functional levels. It is fundamental to further define the biology of these *in vivo* occurring human DC subsets to understand and cure pathogenic immune-mediated processes in so different settings as autoimmunity, infections, and cancer. In particular, appropriate targeting of DC subsets by vaccines holds the promise to induce cytotoxic T-cell responses to eradicate persistent intracellular pathogens or tumors.

## Conflict of Interest Statement

Steve Pascolo is the founder and CEO of Miescher Pharma GmbH, a company exploiting the immunostimulating potential of RNA. The remaining co-authors declare that the research was conducted in the absence of any commercial or financial relationships that could be construed as a potential conflict of interest.
